# Understanding Cancer Screening Behavior in South Korea: A Biopsychosocial Approach to Regional Differences

**DOI:** 10.3390/healthcare13060664

**Published:** 2025-03-18

**Authors:** Yoon-Hee Cho, Joohyun Lee

**Affiliations:** 1 Department of Nursing, College of Nursing, Dankook University, Cheonan 31116, Republic of Korea; choyoonhee@dankook.ac.kr; 2 Department of Nursing, College of Nursing, Eulji University, Seongnam 13135, Republic of Korea

**Keywords:** cancer, screening, predictors, biopsychosocial model, KCHS data

## Abstract

**Background/Objectives:** This study aimed to examine regional cancer screening participation rates among South Korean adults aged 40 and over and to identify biological, psychological, and sociocultural factors associated with cancer screening behavior using the biopsychosocial model. **Methods:** This research was a secondary analysis study. Data were obtained from the 2023 Korean Community Health Survey, focusing on adults residing in cities that exhibited the highest and lowest rates of cancer screening. Differences in cancer screening rates by city were visualized using a location-based open service platform. Variables were categorized into biological, psychological, and sociocultural factors, and logistic regression analysis was conducted to ascertain the factors associated with screening participation. **Results:** The cancer screening rate for adults aged 40 or older in 17 metropolitan cities in Korea ranged from 64.9% to 76.0%, and the national average was 70.9%. In the city with the highest screening rate, participation was positively associated with oral health, physical activity, breakfast-eating habits, and past smoking. In the city with the lowest screening rate, higher screening participation correlated with family cohabitation and satisfaction with the social environment. **Conclusions:** Our results suggest that cancer screening participation rates vary across regions and that the factors associated with cancer screening participation differ between regions with the highest and lowest participation rates. These results provide evidence for targeted interventions that take into account regional factors to improve cancer screening rates in South Korea.

## 1. Introduction

Cancer is a leading cause of death worldwide [1], and recent data estimate that approximately 20 million new cancer cases and 9.7 million cancer-related deaths occurred globally in 2022 [2]. The situation in Korea is similar, with cancer being the leading cause of death in 2023, with 166.7 cases per 100,000 people, accounting for approximately 24% of all deaths [2]. The number of deaths from cancer increases with age, with the highest number of deaths from cancer being among Korean adults aged 40 years or older for both men and women [3]. While cancer is a life-threatening disease, early detection significantly increases the success rate of treatment and reduces mortality. Consequently, cancer screening is recommended as a critical preventive health measure [4].

In South Korea, the National Health Insurance Service offers general health examinations and cancer screening programs, targeting chronic diseases and cancers that rank highly among the causes of death [5,6]. Initially, these screening programs were accessible only to low-income groups, but coverage has progressively expanded, providing services to all citizens based on age, health condition, and income level, either at no cost or at a reduced cost [5,6]. Despite this expansion, participation rates remain suboptimal. In 2023, the participation rate for general health examination was 75.9%, while the rate for cancer screening was lower, at 59.8% [7].

In addition, cancer screening rates among Korean adults vary by region [8], which is similar to the results of previous studies that have investigated regional differences in cervical cancer and breast cancer screening [9] and colorectal cancer screening [10]. Previous studies have suggested that these differences may be due to differences in healthcare access, socioeconomic status, and health awareness [11,12,13]. However, some previous studies have not found regional differences in cancer screening [13,14]. Therefore, in order to implement evidence-based interventions to improve screening uptake, it is necessary to identify regional differences in Korea and, if so, accurately identify factors associated with cancer screening.

Cancer screening is part of the public health system implemented in many countries with the goal of improving population health [15]. Although each country is making efforts to increase participation, participation in cancer screening programs is still suboptimal and often shows significant disparities across regions [15,16,17]. Korea is not an exception to this phenomenon. Therefore, an integrated understanding of the various factors related to individual behavior is essential to explain cancer screening behavior and increase participation [15].

The biopsychosocial (BPS) model is widely applied not only in mental health but also in health education, health psychology, and public health [18]. Engel introduced the BPS model to highlight the significance of incorporating psychosocial factors into the determination of health outcomes, thereby emphasizing the BPS model’s role in comprehensive health assessment [19]. The BPS model offers a comprehensive framework for elucidating the multifaceted influences on health behaviors, as individual health behaviors are shaped by life experiences and current psychological and social contexts [18]. While each component can be examined independently, the primary focus is on their interactions [20]. In this perspective, the BPS model is regarded as a suitable framework for understanding cancer screening behavior.

This study aimed to compare cancer screening rates among adults aged 40 years and older across regions in Korea using the BPS model. The study identified factors associated with cancer screening in regions with the highest and lowest screening rates and used geographic information system (GIS) methods to analyze the geographical distribution of these associated factors within each region. The approach adopted in this study is expected to provide foundational data to inform community-based social and policy interventions.

## 2. Materials and Methods

### 2.1. Study Design

This study was a secondary analysis conducted to examine factors associated with cancer screening participation among adults aged 40 and over. The analysis was based on data from the Korean Community Health Survey (KCHS), with the factors under investigation being analyzed according by community region. The KCHS is an annual retrospective survey conducted by local health centers with a view to assessing health-related indicators across different regions in Korea and to support evidence-based public health programs [21].

### 2.2. Research Framework and Variable Selection

In this study, we investigated factors affecting the likelihood of undergoing cancer screening by applying Engel’s biopsychosocial framework [22]. The BPS model systematically considers biological, psychological, and social factors to understand an individual’s health, disease, and health management [22]. Therefore, it is important to distinguish significant biological, psychological, and social factors to understand an individual’s behavior regarding health promotion, such as cancer screening [22]. Based on the BPS model, we classified potential determinants affecting cancer screening behavior into the following areas through previous studies on health promotion behavior and health screening: (1) biological factors: general characteristics and health status (gender, age, body mass index (BMI), perceived physical health, oral health) [23,24,25,26] and health behaviors, such as smoking, drinking, seatbelt use, physical activity, breakfast consumption, food choices according to nutrition labels, and unmet medical needs [25,27,28]; (2) psychological factors, including stress levels, depression, and addictions [29,30]; and (3) sociocultural factors, including family-related factors (family composition and household income) [26], and community-related factors (satisfaction with the physical and social environment) [31].

### 2.3. Participants

This study examined regional disparities in cancer screening rates across administrative districts in Korea, with a particular focus on data from two cities exhibiting the highest and lowest rates. Using data from the 2023 KCHS, the analysis targeted adults aged 40 and over in each region, based on the minimum age of 40 recommended by Korea’s cancer screening program guidelines [6]. City U and City G are two of the six metropolitan cities in South Korea and are large cities. Generally, a metropolitan city in South Korea can be designated as such when its population exceeds 1 million and it operates independently from the surrounding cities. As of 2022, City U had a population of approximately 1.11 million and City G has a population of approximately 1.46 million. The area of the two regions is 1057 km^2^ for City U and 500.9 km^2^ for City G, and both cities are divided into five administrative districts (gu). In both cities, the original study population was adults aged 19 years and older, and data were collected based on 900 people per administrative unit (gu). Of these, the study sample comprised 6752 participants: 3272 from City G, which had the highest screening rate, and 3480 from City U, which had the lowest screening rate.

### 2.4. Data Collection and Research Ethics

The data for this study were obtained from the 2023 KCHS, which forms part of an annual survey series that has been conducted since 2008. The 2023 KCHS targeted Korean residents aged 19 and over, with trained interviewers collecting data through one-to-one interviews. The survey content included both individual and family questionnaires, with the family questionnaire completed by a single representative from each household.

Prior to the provision of data access online, researchers submitted data use agreements and research proposals to the Korea Disease Control and Prevention Agency (KCDC), which conducted a review of these prior to approval. The data were restricted to the researchers involved in the study and managed to prevent unauthorized access. Additionally, all data provided contained only de-identified information, in compliance with the usage guidelines set by the KCDC. The study was conducted following the approval of an IRB exemption (IRB no. EUIRB2024-109) granted by the researchers’ affiliated institution.

### 2.5. Tools

#### 2.5.1. Biological Factors

The investigation of biological factors was conducted through a comprehensive categorization process, informed by individuals’ general characteristics and health status and health behaviors. The general characteristics and health status of the participants were assessed through a range of variables, including gender, age, the BMI calculated from the self-reported height and weight, the perceived health status, and the perceived oral health status. Both the health and oral health statuses were measured on a scale from 1 to 5, with higher scores indicating better health conditions. The raw data were then recoded to align with the objectives of the study.

The assessment of health behaviors encompassed a range of factors, including smoking, alcohol consumption, seatbelt use, physical activity, breakfast consumption, nutrition label inspection during food selection, and unmet medical needs. Smoking status was categorized into non-smokers, former smokers, and current smokers based on the lifetime smoking status and questions regarding current smoking, including tobacco and e-cigarette use. Alcohol consumption was categorized into three groups based on the frequency of drinking in the past year: non-drinkers, those who drank once a week or less, and those who drank more than twice a week. Seatbelt use was assessed by enquiring whether individuals wore seatbelts while seated in the back seat of a car driven by someone else; individuals who did not ride in others’ cars were excluded from the study. Physical activity was evaluated by the number of days in the past week that participants engaged in moderate-intensity physical activity for at least 10 min, categorized as fewer than three days or three days or more. The regularity of breakfast consumption was determined by asking how frequently participants had breakfast in the previous year, with responses categorized into three or more days and fewer than three days per week. Participants were also asked whether they used nutrition labels when purchasing processed foods. Furthermore, unmet medical needs were identified based on past-year experiences of individuals needing medical services, tests, or treatments for various reasons yet not receiving them.

#### 2.5.2. Psychological Factors

The assessment of psychological factors encompassed the evaluation of subjective stress levels, symptoms of depression, and indicators of addiction. Subjective stress levels were measured on a four-point scale ranging from “I rarely feel stressed in daily life” to “I feel very stressed”, with higher scores denoting heightened stress levels. Depression was gauged based on the presence of feelings of sadness or hopelessness that hindered daily function for a duration exceeding two weeks within the previous year. Finally, addiction was gauged by enquiring whether participants faced disruptions in their daily lives due to activities such as internet use, gaming, smartphone use, or gambling in the past year. All questions were asked in such a way that participants answered on their own, sharing their thoughts and experiences.

#### 2.5.3. Sociocultural Factors

A comprehensive examination of sociocultural factors was conducted, encompassing both family-related and community-related aspects. The family-related factors included the composition of the family unit and the household income. The family composition was categorized into single-person households and households comprising two or more individuals. The household income was stratified based on the average household income in Korea, with a cutoff of KRW 5 million per month [32].

The community-related factors encompassed satisfaction with the physical and social environments of the residential area. Satisfaction with the physical environment was measured through five questions regarding safety levels, the natural environment, living conditions, public transportation availability, and healthcare services. Responses were recorded as “yes” or “no”, with “yes” counted as one point for each question. Social environment factors focused on trust in neighbors and the extent of mutual assistance among community members, and these were also measured using “yes” or “no” responses. In both cases, higher scores indicated greater satisfaction with the physical and social environments.

### 2.6. Statistical Analysis

The analysis was conducted using IBM SPSS version 28.0 (IBM, Armonk, NY, USA). Cancer screening rates were examined across Korea’s 17 metropolitan cities and visualized using SGIS 2024 software, a location-based open service platform [33]. Cities with the highest and lowest cancer screening rates were selected, weighted based on the analysis method in the 2023 KCHS raw data usage guideline, and analyzed reflecting the complex sample design [34]. The independent variables of the study participants from each city were reported by unweighted frequency and weighted percentage, mean, and standard deviations. Differences in independent variables between the two regions were assessed using independent *t*-tests and Rao–Scott chi-squared tests. In addition, binary logistic regression analysis was performed to identify factors associated with the cancer screening behavior in each city, and odds ratios and 95% confidence intervals were reported.

## 3. Results

### 3.1. Cancer Screening Rates by Region

**Figure 1:**
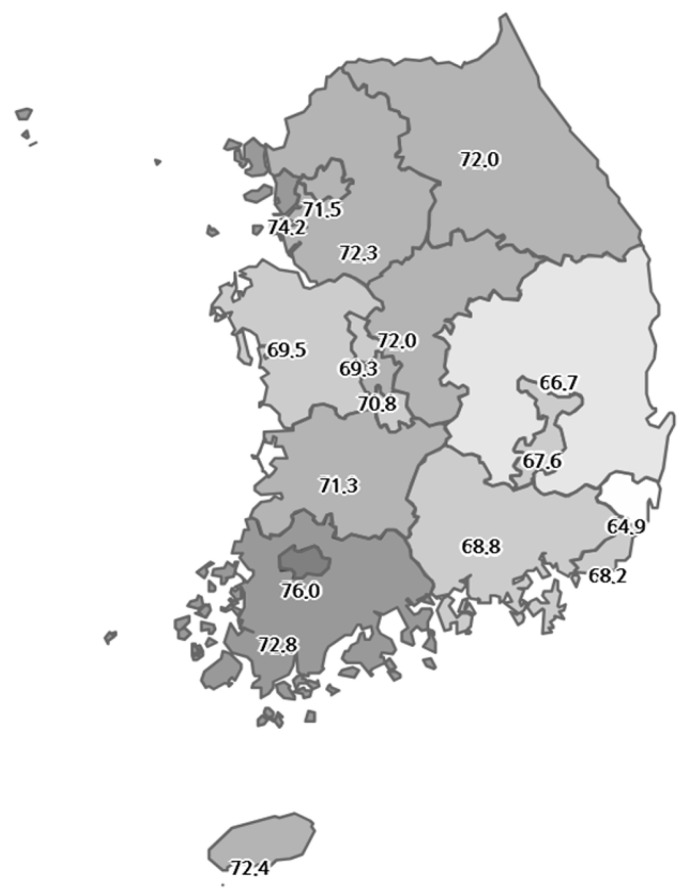
National distribution of cancer screening rates.

The predicted cancer screening rates across South Korea’s 17 metropolitan cities (Table 1) ranged from 51.7% to 62.5% when considering all age groups, with a national average of 57.9%. However, when focusing on adults aged 40 and older, the rates increased, ranging from 64.9% to 76.0%, with a national average of 70.9%. A geographical variation in screening rates is evident in Figure 1, which illustrates that the regions exhibiting the lowest and highest rates were located at a considerable distance from each other.

### 3.2. Biological, Psychological, and Sociocultural Factors in Cities with the Highest and Lowest Cancer Screening Rates

The psychological and sociocultural characteristics of the participants from the two cities are summarized in Table 2. Of the participants, 48.5% were male, with a mean age of 57.96 years, and the mean BMI was 23.64. The mean scores for perceived health and perceived oral health were 3.20 and 2.88 out of 5, respectively. With regard to smoking status, 57.4% of the participants were non-smokers, while 19.9% were current smokers. Alcohol consumption patterns indicated that 47.3% of the participants drank once per week or less, and 33.1% were non-drinkers. The prevalence of seatbelt use in the back seat of an automobile driven by another individual was 39.0%, while the proportion of participants with unmet medical needs for various reasons in the past year was 6.0%. Concerning health behaviors, 23.9% of the participants engaged in moderate-intensity physical activity at least three times per week, and 76.3% reported consuming breakfast at least three times weekly. The average perceived stress level was 2.06 out of 4, with 8.4% of the participants reporting experiences of depression and 7.0% reporting addiction-related issues within the past year. In terms of sociocultural factors, 84.6% of the participants lived with two or more family members, and 38.2% reported a monthly household income of at least KRW 5 million. Satisfaction with the physical environment was rated 3.99 out of 5, while satisfaction with the social environment was rated 0.98 out of 2.

Differences in biological factors between participants from the two cities were observed in gender, the BMI, smoking behavior, drinking behavior, and nutrition label use. Furthermore, disparities in psychological factors were identified, including addiction experiences. Additionally, differences in sociocultural factors were observed, encompassing the family structure, family income, and satisfaction with both the physical and social environments.

### 3.3. Factors Associated with Cancer Screening Behavior in Cities with the Highest and Lowest Cancer Screening Rates

**Figure 2:**
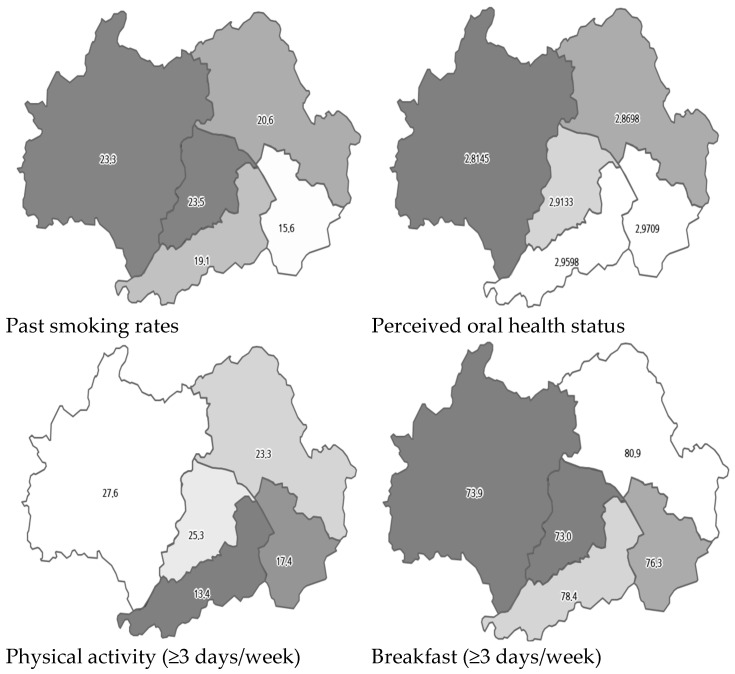
Distribution of associated factors in City G.

As illustrated in Table 3, the findings of the logistic regression analysis revealed the factors associated with cancer screening among adults aged 40 and over in the two cities. It is important that the factors associated with cancer screening participation exhibited variation between the cities. In City G, which had the highest cancer screening rates, men exhibited a lower propensity for cancer screening compared to women (OR: 0.653, 95% CI: 0.49–0.86). Furthermore, the probability of undergoing cancer screening increased with better oral health status (OR: 1.314, 95% CI: 1.17–1.47). In addition, past smokers exhibited higher odds of undergoing cancer screening in comparison to non-smokers (OR: 1.814, 95% CI: 1.28–2.57). Moreover, individuals engaging in moderate-intensity physical activity three or more times per week, as well as those who consumed breakfast at least three times per week, demonstrated higher odds of cancer screening (OR: 1.297, 95% CI: 1.01–1.67; OR: 1.386, 95% CI: 1.08–1.78). The distribution of these related factors within City G is shown in Figure 2.

**Figure 3:**
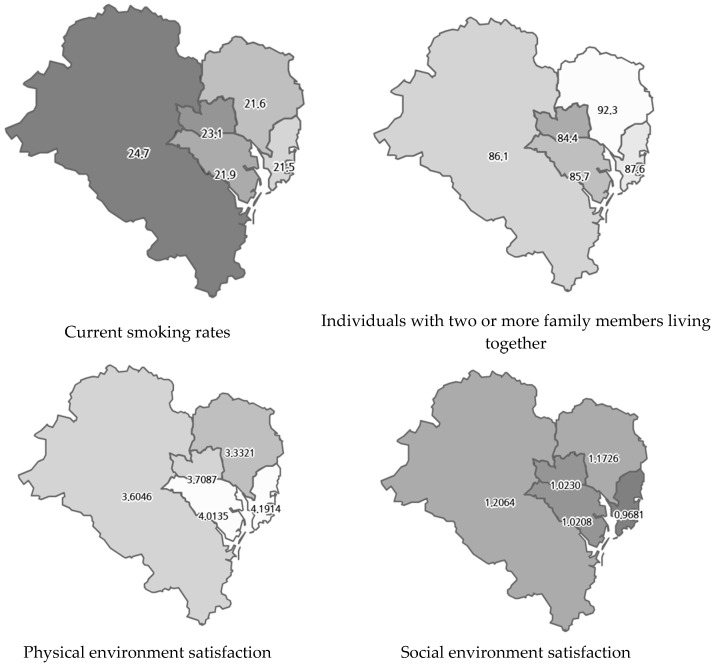
Distribution of associated factors in City U.

In City U, which exhibited the lowest cancer screening rates, current smokers demonstrated a lower propensity to participate in cancer screening compared to non-smokers (OR: 0.506, 95% CI: 0.35–0.73). Moreover, individuals living with family members exhibited higher screening rates compared to those living alone (OR: 1.587, 95% CI: 1.22–2.07). Furthermore, the study identified an association between satisfaction with the physical and social environments and participation in cancer screening. Specifically, lower satisfaction with the physical environment was associated with higher odds of screening (OR: 0.867, 95% CI: 0.80–0.94), while higher satisfaction with the social environment was associated with increased screening likelihood (OR: 1.171, 95% CI: 1.02–1.35). The distribution of these related factors within City U is shown in Figure 3.

## 4. Discussion

This study aimed to investigate the rates of cancer screening participation among South Korean adults aged 40 and over across various geographical regions and to identify the factors influencing screening behavior using the biopsychosocial model. The results revealed significant regional differences in cancer screening participation rates, ranging from 64.9% to 76.0%. Previous studies on geographic differences in cancer screening include those that have found geographic differences [8,9,10] and those that have not found geographic differences [13]. Studies that have found regional differences have reported that the differences are caused by problems in access to medical services and differences in the socioeconomic status between rural and urban areas [11,12]. However, in this study, the regions with the highest and lowest screening rates were both autonomous metropolitan cities with similar geographical conditions. Therefore, more detailed associated factors need to be identified. In this study, the factors influencing screening participation varied between the cities with the highest and lowest participation rates. The findings suggest that public health center staff in local communities should identify factors related to cancer screening participation in order to accurately understand the reasons behind non-participation in cancer screening within the community.

In the city with the highest cancer screening participation rate, factors such as oral health, physical activity, breakfast habits, and past smoking history were all associated with higher participation. This finding aligns with the conclusion of a previous study that identified a correlation between healthy dietary habits, physical activity motivation, and cancer screening participation [35]. A similar trend has been observed in Lithuania, where factors such as the consumption of fresh vegetables, physical activity, and abstinence from alcohol are associated with higher participation in the national breast cancer screening program for women aged 50–69 [36]. In addition, a previous study of cancer screening activity in adults aged 50 years and older in the United States reported that healthy lifestyles, such as physical activity and smoking cessation, are associated with cancer screening behaviors [27]. That is, healthy behaviors are strongly associated with preventive behaviors, such as cancer screening. The findings of this study suggest that promoting healthy behaviors may increase the likelihood of cancer screening participation, and they highlight the need for healthcare providers to provide more proactive health education and to encourage screening among those who exhibit unhealthy behaviors.

Non-smoking or smoking behavior has been identified as a factor influencing cancer screening participation in previous studies [29,35,37]. In this study, past smokers exhibited a higher propensity to participate in cancer screenings, a phenomenon that can be attributed to the use of smoking duration and quantity as indicators for lung cancer screenings in national programs [6]. That is, the Korean government uses adults aged 54 years or older with a smoking history of 30 pack-years or more as a strong recommendation for lung cancer screening programs [6] and provides strong health education on the relationship between smoking and lung cancer. Therefore, the proportion of male smokers, which was high in the past, is decreasing [38]. This situation may determine the association between smoking behavior and cancer screening in Korea. Therefore, it is important to take measures to encourage smokers to quit smoking, and it suggests that encouraging smokers to adopt healthy habits and increase awareness of health risks may increase participation in preventive measures, such as cancer screening.

In City G, men were less likely to participate in cancer screening than women. In general, healthy behaviors, such as cancer screening, often show different patterns by age and gender [39]. Previous studies have also mainly reported gender differences in cancer screening participation [40]. However, some studies have not found an association between gender and lung cancer screening [37]. Previous studies that have found gender differences have mainly reported that better preventive behaviors, such as cancer screening participation, are performed more frequently by women [41]. A possible explanation for this may be that women are exposed to more information than men, as breast and cervical cancer screening encourages cancer screening at a younger age [41]. However, unlike in City G, this study did not observe statistically significant gender differences in City U. Previous studies have shown that cancer screening is associated with various demographic factors, such as age, education level, occupation, and economic status, in addition to gender [27,35]. Since this study did not capture all of these characteristics, caution is needed when interpreting the results. Therefore, future studies should strive to more accurately capture cancer screening behaviors by regional characteristics and gender differences.

In regions exhibiting low participation rates of cancer screening, factors beyond biological considerations also exerted an influence on participation. In City U, which exhibited the lowest screening participation rate, smoking, family composition, and sociocultural factors were associated with reduced participation. Current smokers exhibited a significantly lower likelihood of participating in cancer screenings, underscoring the importance of targeted health education and screening encouragement for populations with unhealthy behaviors.

This study also identified that individuals living with family members or those with higher social environment satisfaction are more inclined to participate in cancer screenings. Social environment satisfaction, encompassing relationships with neighbors, emerged as an important factor. This finding aligns with the results of a previous study that reported that marital status was associated with participation in a colorectal cancer screening program [40]. The results of this study imply that strategies aimed at enhancing cancer screening participation should not only address individuals but also consider the role of family and community involvement.

An important implication of this study is the observation that factors influencing cancer screening participation not only vary across regions but also are influenced by multiple factors. This highlights the need for tailored interventions that take into account regional characteristics, even within the same country. That is, although Cities G and U have similar population sizes and characteristics within the same country, the factors that influence actual cancer screening participation and participation behaviors are different. Therefore, public health providers who manage decisions and programs to increase cancer screening participation in each region need to identify regional influencers to recognize non-participant groups or develop participation programs that take into account relevant factors.

The BPS model used in this study provides a useful framework for understanding individuals’ cancer screening behaviors. In both high- and low-participation regions, factors beyond physiological indicators, particularly health behaviors and family- and community-related factors, played a role and contributed to explaining cancer screening behaviors. Therefore, to increase cancer screening participation, it is essential to adopt a more comprehensive approach that goes beyond simply providing screening services and considers individuals’ physiological, psychological, and social characteristics.

This study has several limitations. First, due to the constraints of secondary data analysis, we could not investigate all the factors considered in previous studies. In particular, there were limitations in the selection of psychological variables that affect individual behavior, and it was difficult to investigate regional differences more deeply. Therefore, future studies should consider the characteristics of community access to healthcare and examine factors affecting cancer screening participation in more detail. Second, since this study analyzed existing data, it is difficult to exclude secondary data bias. Moreover, despite the fact that the present study concentrated on cities with the highest and lowest screening rates, there is a possibility of potential sampling bias due to unmeasured demographic, socioeconomic, or health infrastructure disparities between regions. Additionally, due to the fact that the data collection was dependent on a fixed sample size per administrative district, the representation of certain subgroups within each district may be constrained, which may have an effect on the generalizability of the results. Finally, given the cross-sectional nature of the study, it is difficult to establish a causal relationship, so caution is required when interpreting the results as a causal relationship. Therefore, in future studies, we propose an experimental study that applies the influencing factors suggested in this study to a cancer screening program in the community.

## 5. Conclusions

This study used a biopsychosocial model to investigate cancer screening participation among Korean adults aged 40 years and over living in a large city, exploring the biological, psychological, and sociocultural factors associated with it. An analysis of data from the 2023 Korean Community Health Survey revealed that cancer screening participation rates exhibit variation by region, with the factors associated with the highest and lowest participation rates differing by region. These findings underscore the necessity for a regional biopsychosocial approach in the formulation of interventions to enhance cancer screening participation in Korea.

## Figures and Tables

**Table 1:** Cancer screening rate by region in Korea (17 metropolitan cities, patients over 40 years old).

City	1	2	3	4	5	6	7	8	9	10	11	12	13	14	15	16	17
All ages (%)	56.3	56.8	54.5	60.2	60.9	56.3	51.7	53.6	58.4	60.9	59.8	58.1	59.9	62.5	56.9	57.5	59.6
Age ≥ 40 (%)	71.5	68.2	67.6	74.2	76.0	70.8	64.9	69.3	72.3	72.0	72.0	69.5	71.3	72.8	66.7	68.8	72.4

The city name is anonymous; 5. City G, 7. City U.

**Table 2:** Characteristics of the participants of two cities.

Variable	Total (n = 6752)	City G (n = 3272)	City U (n = 3480)	*t* or Rao–Scott χ^2^ (*p*)
	M ± SE/Unweighted Frequency (Weighted Frequency, %)			
Cancer screening (yes)	4693 (71.0)	2452 (76.0)	2241 (64.9)	57.448 (<0.001)
Biological factors: general characteristics and health status				
Gender (male)	3081 (48.5)	1486 (47.6)	1595 (49.6)	5.262 (0.022)
Age (year)	57.96 ± 0.19	58.25 ± 0.26	57.59 ± 0.26	1.804 (0.072)
BMI	23.64 ± 0.04	23.51 ± 3.05	23.74 ± 3.05	−2.613 (0.009)
Perceived health status	3.20 ± 0.01	3.18 ± 0.88	3.17 ± 0.83	−0.069 (0.945)
Perceived oral health status	2.88 ± 0.01	2.89 ± 0.86	2.84 ± 0.86	0.923 (0.356)
Biological factors: health behaviors				
Smoking (non-smoking)	4059 (57.4)	2101 (61.0)	1961 (53.0)	23.247 (<0.001)
Smoking (past smoking)	1473 (22.7)	697 (21.3)	896 (24.4)	
Smoking (current smoking)	1220 (19.9)	474 (17.7)	623 (22.6)	
Drinking (non-drinking)	2473 (33.1)	1295 (35.4)	1178 (30.2)	9.993 (<0.001)
Drinking (less than once a week)	3057 (47.3)	1438 (46.6)	1619 (53.2)	
Drinking (more than twice a week)	1222 (19.6)	539 (18.0)	683 (21.5)	
Wearing seat belt (yes)	2464 (39.0)	1229 (39.9)	1235 (37.8)	2.153 (0.143)
Physical activity (≥3 days/week)	1547 (23.9)	687 (22.8)	860 (25.2)	3.673 (0.056)
Breakfast (≥3 days/week)	5243 (76.3)	2536 (76.7)	2707 (75.8)	0.522 (0.470)
Reading and using processed food nutrition labels (yes)	1568 (24.2)	678 (22.2)	890 (26.5)	13.313 (<0.001)
Unmet medical care (yes)	356 (6.0)	166 (6.1)	190 (5.9)	0.079 (0.778)
Psychological factors				
Perceived stress status	2.06 ± 0.01	2.02 ± 0.73	2.02 ± 0.75	1.707 (0.088)
Depressive experience (yes)	558 (8.4)	255 (8.4)	303 (8.5)	0.049 (0.826)
Addiction experience (yes)	440 (7.0)	179 (6.2)	261 (8.1)	5.751 (0.017)
Sociocultural factors: family-related				
Family structure (single person)	1103 (15.4)	585 (17.1)	494 (12.8)	16.133 (<0.001)
Family structure (≥two people)	5649 (84.6)	2687 (82.9)	2986 (87.0)	
Family income (<5 million KRW)	3933 (61.8)	1888 (58.7)	2045 (66.3)	12.667 (<0.001)
Family income (≥5 million KRW)	2046 (38.2)	1190 (41.3)	856 (33.7)	
Sociocultural factors: community-related				
Physical environment satisfaction	3.99 ± 0.02	4.26 ± 1.14	3.80 ± 1.35	9.506 (<0.001)
Social environment satisfaction	0.98 ± 0.01	0.96 ± 0.70	1.10 ± 0.79	−7.678 (<0.001)

KRW, Korean won.

**Table 3:** Factors associated with the cancer screening behavior in the two cities.

Variable	City G		City U	
	OR	95% CI	OR	95% CI
Biological factors: general characteristics and health status				
Gender (male)	0.653	0.49–0.86	0.886	0.62–1.22
Age (year)	0.997	0.98–1.01	1.009	0.99–1.02
BMI	1.003	0.97–1.04	1.016	0.98–1.05
Perceived health status	0.917	0.80–1.05	1.052	0.92–1.21
Perceived oral health status	1.314	1.17–1.47	1.074	0.95–1.21
Biological factors: health behaviors				
Smoking (past smoking)	1.814	1.28–2.57	1.001	0.72–1.40
Smoking (current smoking)	0.839	0.60–1.18	0.506	0.35–0.73
Drinking (less than once a week)	0.975	0.76–1.26	1.068	0.84–1.36
Drinking (more than twice a week)	0.901	0.66–1.23	0.997	0.73–1.36
Wearing seat belt (yes)	1.101	0.88–1.38	1.196	0.97–1.48
Physical activity (≥3 days/week)	1.297	1.01–1.67	1.089	0.84–1.41
Breakfast (≥3 days/week)	1.386	1.08–1.78	1.251	0.95–1.64
Reading and using processed food nutrition labels (yes)	0.930	0.70–1.24	1.194	0.92–1.55
Unmet medical care (yes)	0.799	0.52–1.23	0.865	0.58–1.28
Psychological factors				
Perceived stress status	0.977	0.84–1.14	1.018	0.87–1.19
Depressive experience (yes)	1.102	0.76–1.61	0.724	0.49–1.06
Addiction experience (yes)	1.256	0.79–1.99	1.099	0.74–1.63
Sociocultural factors: family-related				
Family structure (≥two people)	1.279	0.99–1.66	1.587	1.22–2.07
Family income (≥5 million KRW)	1.329	0.99–1.77	1.085	0.83–1.42
Sociocultural factors: community-related				
Physical environment satisfaction	1.038	0.94–1.14	0.867	0.80–0.94
Social environment satisfaction	0.994	0.85–1.16	1.171	1.02–1.35

Reference: gender, female; smoking, non-smoking; drinking, non-drinking; wearing seatbelt, no; physical activity, <3 days/week; breakfast, <3 days/week; reading and using processed food nutrition labels, no; unmet medical care, no; depressive experience, no; addiction experience, no; family structure, single person; family income, KRW 5 million.

## Data Availability

The original contributions presented in this study are included in the article. Further inquiries can be directed to the corresponding author.
